# Computed Tomographic Thickness of Retrobulbar Optic Nerve Is Decreased in Glaucoma Patients and Is Negatively Correlated With Disease Severity

**DOI:** 10.7759/cureus.31066

**Published:** 2022-11-03

**Authors:** Cem Evereklioglu, Duygu Gulmez Sevim, Fatih Horozoglu, Osman A Polat, Hidayet Sener, Abdulhakim Coskun, Ahmet Ozturk

**Affiliations:** 1 Department of Ophthalmology, Erciyes University Medical Faculty, Kayseri, TUR; 2 Department of Radiology, Erciyes University Medical Faculty, Kayseri, TUR; 3 Department of Biostatistics, Erciyes University Medical Faculty, Kayseri, TUR

**Keywords:** radiology, retrobulber, optic nerve-sheath complex, glaucoma, computed tomography

## Abstract

Purpose

The purpose of the study was to quantitatively measure the width of the retrobulbar optic nerve-sheath complex by computed tomography (CT) in glaucoma and evaluate its relationship with optic nerve changes and visual field loss parameters.

Methods

Sixty-six eyes of 33 patients with bilateral asymmetric glaucomatous optic nerve damage and 20 eyes of 20 age- and sex-matched control subjects without glaucoma were included. Axial retrobulbar optic nerve-sheath complex was measured by CT in the eyes with advanced glaucomatous damage (group 1); in the fellow eyes of the same patients with moderate glaucomatous damage (group 2); and in control subjects (group 3). Measurements were obtained at three different points: just behind the globe, near the optic canal, and in the middle.

Results

Mean age and sex distribution between groups were comparable (p>0.05). The mean diameters of the retrobulbar optic nerve at three measurement points in group 1 (4.00±0.42mm, 3.49±0.44mm, 3.18±0.45mm) were significantly (for each, *p*<0.05) lower when compared with the corresponding points of group 2 eyes (4.24±0.41mm, 3.77±0.47nn, 3.47mm±0.44mm) and normal controls (4.58±0.44mm, 4.15±0.45mm, 3.92±0.48mm). Optic disc changes and visual field parameters were negatively correlated with retrobulbar optic nerve diameter (for each, *p* < 0.05).

Conclusion

Radiological alterations of the retrobulbar optic nerve in glaucomatous eyes revealed a decreased optic nerve diameter which correlated with disease severity. Optic nerve dimensions below the lower limit for normal individuals may be considered pathologically reduced and, therefore, CT measurements of the retrobulbar nerve may be additive to the traditional triad of raised intraocular pressure, field defects, and optic disc changes in some cases with opaque optic media preventing the fundus examination or with optic nerve anomalies.

## Introduction

Glaucoma is defined as a progressive optic neuropathy characterized by optic nerve head excavation and glaucomatous visual field loss, enclose mechanical and vasogenic mechanisms [1, 2]. Optic nerve head changes in glaucoma include neuroretinal rim loss, optic cup deepening and widening with localized or diffuse loss of the retinal nerve fiber layer (RNFL) [3]. Attention has focused on the optic disc shape and volume with computerized techniques [4]. These morphological optic disc alterations can be divided into two groups. Qualitative criteria such as the presence of neuroretinal rim notches, disc hemorrhages, and RNFL defects are mostly used to demonstrate the presence of glaucomatous damage of the optic nerve [5, 6]. RNFL thickness, neuroretinal rim area, and the measurement of optic cup volume consist, in turn, of the quantitative variables [7].

Investigators have reported several promising methods for the detection of glaucoma, including scotopic sensitivity test, peripheral color contrast, and interocular brightness sense test [8, 9]. Currently used sophisticated methods include optic nerve head photography, optic nerve head analyzer, optic disc morphometry, optical coherence tomography, and confocal scanning laser ophthalmoscopy, all of which evaluate the optic nerve head or RNFL [10, 11].

Computed tomography (CT) has become a widely used technique for imaging of the orbit since the 1970s. Although we have a classic knowledge about the optic disc shape, visual field defects, and RNFL damage clinically, the size of retrobulbar optic nerve-sheath complex in the course of glaucoma remains to be specified. In other words, if we had been asked about the size of the retrobulbar optic nerve and the influence of glaucomatous optic disc atrophy on it, we would not have been able to give numbers.

Therefore, this study aimed at (1) gathering further objective and quantitative radiological data about the retrobulbar segment of the orbital optic nerve in patients with glaucoma compared to those of age-adjusted controls, and (2) evaluating the possible correlations between the sizes and cup:disc ratio of these eyes.

## Materials and methods

Patients

Because the CT examination is invasive imaging in terms of radiation, the glaucoma patients were selected from those who had also some other CT indications from paranasal sinus diseases or such. The study conformed to the tenets of the Declaration of Helsinki and informed consent was obtained or waived by all participants after the study was approved by theInstitutional Review Board (IRB) of Erciyes University Faculty of Medicine Department’s Board (approval number 011122/62).

A total of 66 eyes of 33 consecutive Caucasoid patients with bilateral but asymmetric glaucomatous optic nerve damage (17 men, 16 women) and 20 eyes of 20 age- and sex-matched control subjects without glaucoma (10 men, 10 women) were enrolled in this cross-sectional study. Patients with glaucoma were admitted to our clinic for uncontrolled intraocular pressure (IOP) and progressive glaucomatous optic disc damage with visual field loss despite maximal tolerated medical therapy. All patients underwent a complete ophthalmic examination including slit-lamp biomicroscopy. Entry criteria were classical glaucomatous optic nerve damage associated with visual field loss in the corresponding hemifield location on the 30-2 Humphrey visual field analyzer standard (Humphrey Systems, Inc., Dublin, California, USA), glaucomatous optic nerve head damage with the cup:disc ratio over 0.4, the presence of excavation or undermining of the cup, rim notches, RNFL defects characteristic of glaucoma, disc hemorrhages, or cup:disc asymmetry of greater than 0.2. Central 30-2 full threshold test with the white target was used for visual field analysis. Visual field reliability criteria included less than 25% fixation losses and false-positive and false-negative rates [12,13]. Patients were divided into two groups according to laterality; the eyes with advanced glaucomatous optic nerve damage (group 1, n = 33); and the fellow eyes of the same patients with moderate glaucomatous optic nerve damage (group 2, n = 33).

Control subjects without glaucoma were those referred to the Department of Radiology with the indications of CT for suspicion of tumor or tumor-like conditions of the paranasal sinuses, or CT indication for migraine-like symptoms. All subjects were free of clinical evidence or history of endocrine disease, orbital disorder or any other systemic disease affecting the eye. Additional criteria were - best corrected visual acuity of 20/20; IOP less than 21 mm Hg as measured by Goldmann applanation tonometry; no significant ocular disease found in routine eye examination; spherical equivalent refractive status within ± 2.00 dioptres (D); normal funduscopic optic disc appearance; and normal perimetry with no history of glaucoma in the family. Absence of glaucomatous optic neuropathy was defined as a vertical cup:disc asymmetry less than 0.2, cup:disc ratio less than 0.4, and an intact neuroretinal rim without peripapillary hemorrhages, notches, localized pallor, or RNFL defect. In radiological evaluation, asymmetric scans, scans with artifacts for any reason (dental material, eye motion, etc.) that may cause errors in orbit measurements, and CT scans with abnormal orbital findings were excluded. Although we measured both eyes, only the right eye of each control subject (group 3, n = 20) was used for statistics.

Radiological measurement of the optic nerve-sheath complex

Axial 3-mm-thick non-overlapping contiguous sections were obtained from all eyes in glaucoma patients and controls with a CT scanner (Prospeed, General Electric, Milwaukee, USA). Axial scans were obtained at an angle of −10° to −15° relative to the orbitomeatal plane. Patients were asked to maintain a forward gaze and gentle eye closure during the scans to prevent asymmetric extraocular muscle contraction. Because every change in the window settings resulted in different measurements in the same CT scan, all scanning procedures were performed at constant window level and width settings of 50 and 250 H, respectively. To determine the normal globe position in CT, the interzygomatic line at the midglobe section was used as a reference line [12-14]. The width of the optic nerve-sheath complex was measured perpendicular to its course in the axial CT sections. The measurements were obtained in the proximal (near the optic canal), distal (just behind the globe), and in the middle portion of the nerve. In addition, we measured some additional parameters as described before [14] to match the right and left orbits of each subject as well as to compare the eyes of glaucoma patients and control subjects. Therefore, the length of the interzygomatic line and the posterior margin of the globe at the midglobe section were measured on axial scans. Furthermore, the diameters of the lateral and medial rectus muscles were also measured at their maximum. Measurements were performed directly on the magnified hard-copy images with the same magnification factor for all subjects in both groups and were converted to true size in millimeters. The same radiologist performed all measurements masked to the diagnosis or group of the patients and controls.

Statistics

All datasets were subjected to normality testing using the Shapiro-Wilks test and data were expressed as mean ± standard deviation (SD) (for normally distributed data) or as median with range (for skewed data). Continuous variables were analyzed with parametric or non-parametric methods as indicated according to whether they showed normal distribution or not. Kruskal-Wallis Variance Analysis (multiple comparisons were carried out with Dunn’s test) and One Way Analysis of Variance (multiple comparisons were carried out with Bonferroni test) tests were used to compare each variable between the groups. Correlation analysis was performed by Spearman’s correlation coefficient to assess associations between optic nerve changes and the visual field loss parameters for each group. A p-value less than 0.05 was considered to be significant. All statistics in the present study were performed using SPSS for Windows (Version 22.0, IBM Corp., Armonk, New York, USA).

## Results

Mean values for the length of the interzygomatic line, the diameters of the measured extraocular muscles, and the distance between the interzygomatic line and the posterior margin of the globe did not show a statistically significant difference between data for the right and left orbits of all subjects in both groups (for each, p > 0.05).

Right eyes were used for statistical analysis in control subjects. The mean age between the patients (66.86 ± 4.34 years, [range, 62-76]) and control subjects (65.75 ± 4.17 years, [range, 60-74]) was comparable (p > 0.05). All patients who participated in the present study had primary open-angle glaucoma with an open anterior chamber angle, raised IOP (measurements above 22 mm Hg), and abnormal optic nerve head with glaucomatous visual field defects in the perimetric examination.

The mean spherical equivalent refractive error of group 1, group 2, and group 3 eyes (-0.43 ± 0.56 D, -0.39 ± 0.51 D, and -0.41 ± 0.49 D, respectively) was similar (p > 0.05). Best-corrected visual acuity by the Snellen chart was below 20/200 in all eyes with advanced glaucomatous damage. The fellow eyes with a moderate glaucomatous damage had visual acuity between 20/200 and 20/20. Mean preoperative IOP (despite maximal tolerated medical therapy) was 30.63 ± 3.20 mm Hg in group 1, 22.42 ± 1.54 mm Hg in group 2, and 15.85 ± 1.66 mm Hg in normal control subjects.

The mean diameter of the retrobulbar optic nerve-sheath complex in group 1 eyes (mean cup:disc ratio; 0.86 ± 0.09) was 4.00 ± 0.42 mm just behind the globe, decreasing to 3.49 ± 0.44 mm in the middle portion, and 3.18 ± 0.45 mm near the optic canal. These values were significantly lower when compared to the corresponding points of the fellow group 2 eyes (mean cup:disc ratio; 0.56 ± 0.08) of the same patients or control subjects (Table 1).

**Table 1:** Radiological and visual function characteristics of glaucomatous eyes and control subjects with statistical analysis a,b,c; Groups with different superscript letters “a, b, and c” were found to have statistically significant (p < 0.05) differences when the comparisons were made between them. Kruskal-Wallis Variance Analysis or One Way Analysis of Variance tests was used as indicated to compare each variable between the groups. CI; confidence interval, CPSD; corrected-pattern standard deviation, IOP; intraocular pressure, MD; mean deviation, RONT; retrobulbar optic nerve thickness.

	Group 1	Group 2	Group 3
	(Advanced Glaucoma)	(Moderate Glaucoma)	(Control Subjects)
	n = 33	n = 33	n = 20
	X ± SD (min-max)	X ± SD (min-max)	X ± SD (min-max)
	(95%CI)	(95%CI)	(95%CI)
Preoperative IOP mm Hg	30.63 ± 3.20^a^ (29.49 - 31.77)	22.42 ± 1.54^b^ (21.87 - 22.97)	15.85 ± 1.66^c^ (15.07 - 16.62)
Cup:disc ratio	0.86 ± 0.09^a^ (0.83 - 0.89)	0.56 ± 0.08^b^ (0.53 - 0.59)	0.23 ± 0.06^c^ (0.19 - 0.26)
MD (dB)	−22.65 ± 11.34^a^ (−26.68 - −18.63)	−9.53 ± 6.54^b^ (−11.85 - −7.21)	−1.02 ± 0.88^c^ (−1.44 - −0.61)
CPSD (dB)	20.12 ± 10.39^a^ (16.44 - 23.81)	8.32 ± 6.11^b^ (6.15 - 10.49)	0.91 ± 0.76^c^ (0.57 - 1.24)
RONT (just behind the globe) mm	4.00 ± 0.42^a^ (3.85 - 4.14)	4.24 ± 0.41^b^ (4.09 - 4.39)	4.58 ± 0.44^c^ (4.37 - 4.79)
RONT (in the middle) mm	3.49 ± 0.44^a^ (3.33 - 3.65)	3.77 ± 0.47^b^ (3.60 - 3.94)	4.15 ± 0.45^c^ (3.93 - 4.36)
RONT (near the optic canal) mm	3.18 ± 0.45^a^ (3.02 - 3.34)	3.47 ± 0.44^b^ (3.31 - 3.63)	3.92 ± 0.48^c^ (3.69 - 4.14)

Group 2 eyes with a moderate level of glaucomatous optic nerve damage also had significantly lower optic nerve thickness when compared with the corresponding points of control subjects. In correlation analysis, retrobulbar optic nerve thickness was significantly (but negatively) correlated with the cup:disc ratio both in group 1 and group 2 eyes (Table 2). Moreover, visual field loss parameters (mean deviation, corrected-pattern standard deviation) were negatively correlated with optic nerve changes (cup:disc ratio) in both group 1 and group 2 (Table 2).

**Table 2:** Correlation analysis* between the cup:disc ratio and retrobulbar optic nerve thickness and visual field loss parameters *Spearman’s correlation coefficient was used as indicated. MD = mean deviation; CPSD = corrected-pattern standard deviation; RONT = retrobulbar optic nerve thickness.

	Group 1		Group 2		Group 3	
	Cup:disc ratio		Cup:disc ratio		Cup:disc ratio	
	r-Value	p-Value	r-Value	p-Value	r-Value	p-Value
RONT (just behind the globe) mm	−.422	.015	−.573	< .001	−.109	.647
RONT (in the middle) mm	−.366	.036	−.484	.004	−.106	.656
RONT (near the optic canal) mm	−.401	.021	−.371	.033	−.366	.113
MD (dB)	−.811	< .001	−.486	.004	−.123	.606
CPSD (dB)	−.819	< .001	−.469	.006	−.191	.420

## Discussion

We evaluated the retrobulbar segment of the orbital optic nerve of 66 eyes with advanced or moderate glaucoma and of 20 controls for the diameter of the optic nerve-sheath complex. As could be expected, the diameter of the optic nerve itself and the retrobulbar optic nerve-sheath complex was significantly reduced in the glaucomatous eyes due to the possible advanced or moderate loss of axons. Our most interesting finding was that the diameter of the retrobulbar optic nerve-sheath complex was both decreased and correlated with the advancement of the glaucomatous damage. Our clinical results were consistent with a morphological study in donors that demonstrated a decreased mean diameter of the optic nerve in the glaucoma group when compared to controls [12]. Although Ozgen and Ariyurek demonstrated that the width of retrobulbar optic nerve-sheath complex was not different between male and female subjects, we still paid particular attention to the gender ratio and mean age between glaucoma patients and controls, since it is known that age affects the number of optic nerve fibers of an individual [13-15].

The criteria for clinical diagnosis and follow-up of glaucomatous damage include the abnormal shape of the neuroretinal rim, high cup:disc ratio, localized or diffuse RNFL defects, and the presence of splinter-shaped hemorrhages at the optic disc border. In addition to these qualitative or semi-quantitative values, there are some quantitative methods that need additional specific hardware and software [16]. Although at present, we evaluate the orbit and extraocular muscles by CT for many reasons such as orbital wall fracture, Graves’s orbitopathy, metastatic tumors, primary neoplasm, vascular malformations, and inflammation, we do not have any numeric results to evaluate objectively the diameter of retrobulbar optic nerve-sheath complex by CT during the course of glaucoma [17, 18].

The retrobulbar optic nerve is the extension of the axons of the retinal ganglion cells passing to the retrobulbar space, which is then myelinated immediately. Some previous studies in humans and experimental models of glaucoma have histologically demonstrated a correlation between the optic nerve neuroretinal rim area and the number of retrobulbar optic nerve fibers [19, 20]. A similar correlation of optic disc size with optic nerve head topographic measurements has also been shown by scanning laser tomography [21]. Likewise, it is known that RNFL thickness is positively correlated with the optic nerve head [22]. Thus, it seems reasonable to examine objectively the thickness of retrobulbar optic nerve-sheath complex by CT and the possible correlation with a clinical finding, the cup:disc ratio. In a study comparing the CT and echography of optic nerve in glaucoma, the authors have found an ample correlation between the standardized A-scan echography and computed axial tomography in both healthy subjects and POAG patients [23]. The study has also demonstrated an important and direct relation between optic nerve diameter reduction and the glaucomatous damage stage. Although echographic A-scan running costs are relatively low and non-invasive, it requires an experienced expert radiologist to read and interpret the data, as compared to CT scans which also have the advantage of documenting the optic nerve course.

**Figure 1:**
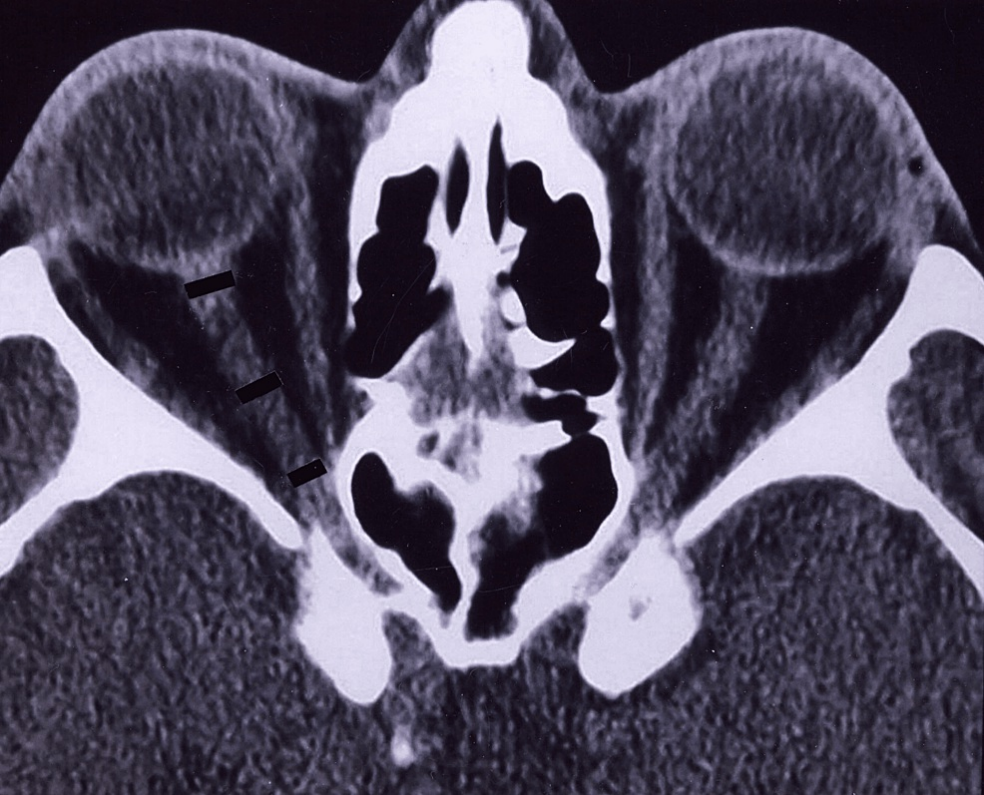
A patient with axial proptosis without glaucoma (normal retrobulbar optic nerve) This figure shows the diameters of the retrobulbar optic nerve by axial computed tomography scan at the midglobe level in a 58-year-old man with bilateral axial proptosis without any clinical sign of glaucomatous optic nerve damage (visual acuities were 20/20 bilaterally, cup:disc ratio was 0.2 in both eyes, and visual field analysis demonstrated normal findings). Both measurements in the middle portion of the nerve (4.88 mm on the right and 5.00 mm on the left) were above the mean values of group 1 (mean, 3.49 mm) and group 2 eyes (mean, 3.77 mm) of glaucoma subjects.

In the present study, the thickness of the retrobulbar optic nerve-sheath complex of glaucomatous eyes showed a significant but negative correlation with the increase of cup:disc ratio. Likewise, in the eyes with advanced glaucomatous damage, the diameter of the retrobulbar optic nerve was significantly thinner than those in the fellow eyes of the same patients with moderate levels of glaucomatous damage and those in control subjects. Such a decrement in the diameter was apparent along all points of the optic nerve behind the globe. Our values in glaucoma subjects were significantly lower when compared with the corresponding mean data of normal control subjects found in this study and than those of normative values of Ozgen and Ariyurek [15]. One of the patients with axial proptosis without glaucoma is shown in Figure 1 for the interpretation of the retrobulbar optic nerve.

**Figure 2:**
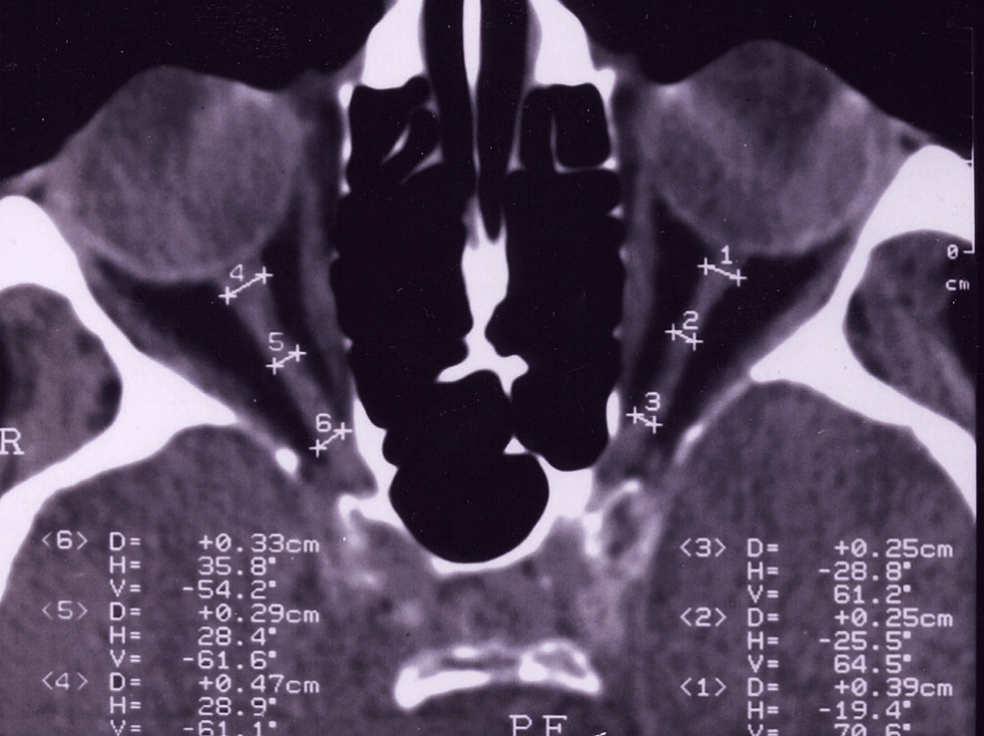
The numeric results for 1st, 2nd, and 3rd retrobulbar points on the left eye were 5.11 mm, 4.88 mm, and 4.70 mm, respectively which were significantly higher when compared with those contralateral glaucomatous eyes This illustration shows a 62-year-old man with bilateral but asymmetric glaucomatous optic nerve damage. The axial diameter of the retrobulbar optic nerve-sheath complex in the middle portion of this patient was 2.90 mm on the right (cup:disc ratio = 0.6) and 2.50 mm on the left (cup:disc ratio = 1.0, total optic atrophy). These measured values for both eyes were clearly thinner than the corresponding mean data of normal control subjects found in this study (mean = 4.15 mm; range 3.50-5.00 mm) and than those of normative values studied before (mean = 4.40 mm; range, 3.20-5.60 mm).

The numeric results of the measured (indicated) 1st, 2nd, and 3rd retrobulbar points on the left eye were 5.11 mm, 4.88 mm, and 4.70 mm, respectively. These values were significantly higher when compared with those in glaucomatous eyes (Figure 2).

The pathology of glaucomatous disease in glaucoma consists of the mechanical hold-up of retrograde and/or orthograde intra-axoplasmic flow from deteriorated intra-axoplasmic pressure gradient after the compression and collapse at lamina cribrosa. Similarly, remodeling of the intraocular portion of the meningeal sheaths in early glaucoma after reactive glial proliferation with increased meningothelial cells in subarachnoid space resulted in the thickening of the pia mater [24]. In addition, vitreal aqueous flow into the cerebrospinal fluid (CSF) space from opened lamina cribrosa pores as a result of optic nerve fiber atrophy after the changed pressure barrier between IOP and CSF pressure has been demonstrated by Jonas et al. [25].

MRI of the brain in infants with congenital glaucoma demonstrated abnormal findings suggesting agenesis of the corpus callosum with delayed myelinization in the white matter in some cases [26]. Similarly, cortical activity was found to be altered in glaucoma subjects in a manner consistent with damage to the optic disc, suggesting functional MRI as a new diagnostic technique for possible means of quantifying cortical neurodegeneration in such cases [27]. Beatty et al. have also reported echographic measurements of the retrobulbar optic nerve in normal and glaucomatous eyes [28]. In addition, Kashiwagi et al. demonstrated that glaucoma affected the anterior visual pathway anterogradely at least up to the optic chiasm that was correlated with glaucomatous optic nerve damage, suggesting MRI of the anterior visual pathway again as a good tool for evaluating glaucomatous damage objectively [29]. Our study also evaluated the anterior visual pathway up to the optic canal and may be used as a novel tool for evaluating glaucomatous damage objectively at least in some selected cases.

There are many ongoing trials that use mesenchymal stem cells and/or their secretome, or plasma, and such for the restoration of optic atrophy in glaucoma [30, 31]. These treatment strategies have less chance to work in the later and more severe stages of the disease. We think that imaging modalities that show the retrobulbar optic nerve may be able to help differentiate the patients in the severe stage of glaucoma who have a better chance to benefit from restorative treatment modalities, as retinal ganglion cell axons project to the central nervous system and their number within the retrobulbar optic nerve may be a suitable surrogate marker for optic atrophy [32]. 

However, this study has some limitations. We do not know whether this method is important for the detection of early glaucomatous optic nerve damage. Likewise, we did not investigate whether rim area, rim volume, and retinal nerve layer thickness were correlated with radiological retrobulbar optic nerve thickness. In addition, we did not assess whether this method was sensitive or specific for glaucoma. However, it is open to further studies on whether this method is reproducible both within and between the examiners. Even though CT evaluation involves radiation exposure, we do not suggest the routine work-up with CT scan for glaucoma patients, rather we suggest that it can be able to help to identify the glaucoma patients that had CT scans for various reasons, and help them get the glaucoma diagnosis. 

Nonetheless, although the cost-benefit relation seems to be important, we think that CT evaluation of the retrobulbar optic nerve thickness is a novel, qualitative and objective method and may be used in cases with severe corneal or lenticular media opacities that prevent the evaluation of posterior segment. Moreover, since the axonal loss is irreversible, it is crucial to be able to determine the patients at risk before the functional loss occurs. The window settings should be the same to accurately compare the sizes both between the different patients and between the different CT examinations of the same patients. Indeed, our values and comparisons between the fellow eyes of the same subjects seem to be valid for specific window level and width settings, 50 and 250 H, respectively.

## Conclusions

It is known that current sophisticated imaging methods such as confocal scanning laser ophthalmoscopy, scanning laser polarimetry, and optical coherence tomography are able to provide objective and quantitative valuables to determine the optic nerve structures for the follow-up and diagnosis of glaucoma to patients that have already applied to the ophthalmology clinics and have the suspicion or diagnosis of glaucoma. However, it is known that patients with glaucoma mainly do not develop any recognizable symptoms until some irreversible anatomical loss is already present. So the main aim of our study was to be able to select the patients by radiologists or other clinicians who might have ordered CT scans for various indications who have the signs of glaucoma and refer the patient’s to the ophthalmologists for glaucoma check. Our results also may help to understand better the physiopathological process and structure-function relationship of glaucoma. We demonstrated for the first time that the width of the retrobulbar optic nerve-sheath complex by radiological CT imaging was significantly thinner in the eyes with glaucoma which was negatively correlated with the advancement of the disease. Such objective and quantitative analyses may demonstrate glaucomatous damage as an alternative approach in patients with opaque optic media from corneal or lenticular disorders.

## References

[1] Evereklioglu C, Doganay S, Er H (2002). Aqueous humor adrenomedullin levels differ in patients with different types of glaucoma. Jpn J Ophthalmol.

[2] Doganay S, Evereklioglu C, Turkoz Y, Er H (2002). Decreased nitric oxide production in primary open-angle glaucoma. Eur J Ophthalmol.

[3] Mardin CY, Horn FK, Jonas JB, Budde WM (1999). Preperimetric glaucoma diagnosis by confocal scanning laser tomography of the optic disc. Br J Ophthalmol.

[4] Sommer A, Pollack I, Maumenee AE (1979). Optic disc parameters and onset of glaucomatous field loss. I. Methods and progressive changes in disc morphology. Arch Ophthalmol.

[5] Pederson JE, Anderson DR (1980). The mode of progressive disc cupping in ocular hypertension and glaucoma. Arch Ophthalmol.

[6] Quigley HA, Addicks EM, Green WR (1982). Optic nerve damage in human glaucoma. III. Quantitative correlation of nerve fiber loss and visual field defect in glaucoma, ischemic neuropathy, papilledema, and toxic neuropathy. Arch Ophthalmol.

[7] Jonas JB, Konigsreuther KA (1994). Optic disk appearance in ocular hypertensive eyes. Am J Ophthalmol.

[8] Yu TC, Falcao-Reis F, Spileers W, Arden GB (1991). Peripheral color contrast. A new screening test for preglaucomatous visual loss. Invest Ophthalmol Vis Sci.

[9] Cummins D, MacMillan ES, Heron G, Dutton GN (1994). Simultaneous interocular brightness sense testing in ocular hypertension and glaucoma. Arch Ophthalmol.

[10] Hepsen IF, Evereklioglu C (2001). Defective visual field tests in chronic heavy smokers. Acta Ophthalmol Scand.

[11] Caprioli J, Prum B, Zeyen T (1996). Comparison of methods to evaluate the optic nerve head and nerve fiber layer for glaucomatous change. Am J Ophthalmol.

[12] Bohdanecka Z, Orgül S, Meyer AB, Prünte C, Flammer J (1999). Relationship between blood flow velocities in retrobulbar vessels and laser Doppler flowmetry at the optic disk in glaucoma patients. Ophthalmologica.

[13] Balazsi AG, Rootman J, Drance SM (1984). The effect of age on the nerve fiber population of the human optic nerve. Am J Ophthalmol.

[14] Mikelberg FS, Yidegiligne HM, White VA (1991). Relation between optic nerve axon number and axon diameter to scleral canal area. Ophthalmology.

[15] Ozgen A, Ariyurek M (1998). Normative measurements of orbital structures using CT. AJR Am J Roentgenol.

[16] Trokel SL, Hilal SK (1979). Recognition and differential diagnosis of enlarged extraocular muscles in computed tomography. Am J Ophthalmol.

[17] Rothfus WE, Curtin HD (1984). Extraocular muscle enlargement: a CT review. Radiology.

[18] Patrinely JR, Osborn AG, Anderson RL (1989). Computed tomographic features of nonthyroid extraocular muscle enlargement. Ophthalmology.

[19] Jonas JB, Schmidt AM, Müller-Bergh JA, Schlötzer-Schrehardt UM, Naumann GO (1992). Human optic nerve fiber count and optic disc size. Invest Ophthalmol Vis Sci.

[20] Caprioli J, Miller JM (1987). Optic disc rim area is related to disc size in normal subjects. Arch Ophthalmol.

[21] Yücel YH, Gupta N, Kalichman MW (1998). Relationship of optic disc topography to optic nerve fiber number in glaucoma. Arch Ophthalmol.

[22] Kee C, Koo H, Ji Y, Kim S (1997). Effect of optic disc size or age on evaluation of optic disc variables. Br J Ophthalmol.

[23] Boles Carenini B, Tettoni E, Brogliatti B (2002). CT and a echography of optic nerve in glaucoma. Acta Ophthalmol Scand Suppl.

[24] Pache M, Meyer P (2006). Morphological changes of the retrobulbar optic nerve and its meningeal sheaths in glaucoma. Ophthalmologica.

[25] Jonas JB, Berenshtein E, Holbach L (2003). Anatomic relationship between lamina cribrosa, intraocular space, and cerebrospinal fluid space. Invest Ophthalmol Vis Sci.

[26] Dai AI, Saygili O (2007). Brain MRI findings in infants with primary congenital glaucoma. Ann Saudi Med.

[27] Duncan RO, Sample PA, Weinreb RN, Bowd C, Zangwill LM (2007). Retinotopic organization of primary visual cortex in glaucoma: a method for comparing cortical function with damage to the optic disk. Invest Ophthalmol Vis Sci.

[28] Beatty S, Good PA, McLaughlin J, O'Neill EC (1998). Echographic measurements of the retrobulbar optic nerve in normal and glaucomatous eyes. Br J Ophthalmol.

[29] Kashiwagi K, Okubo T, Tsukahara S (2004). Association of magnetic resonance imaging of anterior optic pathway with glaucomatous visual field damage and optic disc cupping. J Glaucoma.

[30] Khatib TZ, Martin KR (2020). Neuroprotection in glaucoma: towards clinical trials and precision medicine. Curr Eye Res.

[31] Fang CEH, Guo L, Hill D (2020). Neuroprotective strategies in glaucoma-translation to clinical trials. OBM Neurobiol.

[32] Lagrèze WA, Gaggl M, Weigel M (2009). Retrobulbar optic nerve diameter measured by high-speed magnetic resonance imaging as a biomarker for axonal loss in glaucomatous optic atrophy. Invest Ophthalmol Vis Sci.

